# Molecular determinants of orexin receptor-arrestinubiquitin complex formation

**DOI:** 10.1111/bph.12481

**Published:** 2013-12-23

**Authors:** Werner C Jaeger, Ruth M Seeber, Karin A Eidne, Kevin DG Pfleger

**Affiliations:** 1 Laboratory for Molecular Endocrinology-G Protein-Coupled Receptors Western Australian Institute for Medical Research (WAIMR) and Centre for Medical Research, The University of Western AustraliaPerth, WA, Australia

**Keywords:** BRET, arrestin, GPCR, orexin, hypocretin, ubiquitin

## Abstract

**Background and Purpose:** The orexin system regulates a multitude of key physiological processes, particularly involving maintenance of metabolic homeostasis. Consequently, there is considerable potential for pharmaceutical development for the treatment of disorders from narcolepsy to metabolic syndrome. It acts through the hormonal activity of two endogenous peptides, orexin A binding to orexin receptors 1 and 2 (OX_1_ and OX_2_) with similar affinity, and orexin B binding to OX_2_ with higher affinity than OX_1_ receptors. We have previously revealed data differentiating orexin receptor subtypes with respect to their relative stability in forming orexin receptor-arrestin-ubiquitin complexes measured by BRET. Recycling and cellular signalling distinctions were also observed. Here, we have investigated, using BRET, the molecular determinants involved in providing OX_2_ receptors with greater β-arrestin-ubiquitin complex stability.

**Experimental Approach:** The contribution of the C-terminal tail of the OX receptors was investigated by bulk substitution and site-specific mutagenesis using BRET and inositol phosphate assays.

**Key Results:** Replacement of the OX_1_ receptor C-terminus with that of the OX_2_ receptor did not result in the expected gain of function, indicating a role for intracellular domain configuration in addition to primary structure. Furthermore, two out of the three putative serine/threonine clusters in the C-terminus were found to be involved in OX_2_ receptor-β-arrestin-ubiquitin complex formation.

**Conclusions and Implications:** This study provides fundamental insights into the molecular elements that influence receptor-arrestin-ubiquitin complex formation. Understanding how and why the orexin receptors can be functionally differentiated brings us closer to exploiting these receptors as drug targets.

**Linked Articles:** This article is part of a themed section on Orexin Receptors. To view the other articles in this section visit http://dx.doi.org/10.1111/bph.2014.171.issue-2

## Introduction

The orexin system plays a critical role in maintaining and integrating primordial physiological functions including sleep-wake transitions and metabolic signals controlling energy homeostasis, as well as modulation of addictive behaviour processes and dependencies (Sakurai and Mieda, 2011; Kim *et al*., 2012).

Both orexin receptors (OX_1_ and OX_2_; receptor nomenclature follows Alexander *et al*., 2013) typically couple with the G_q_ subclass of G proteins upon stimulation, resulting in release of inositol phosphates and elevation of intracellular calcium levels (Sakurai *et al*., 1998). In addition, it is evident that both these receptors robustly recruit members of the multi-adaptor protein family, the arrestins (Milasta *et al*., 2005; Dalrymple *et al*., 2011).

The formation of arrestin-bound GPCR complexes can confer a wide range of regulatory and cellular signalling functions to the complex. Activated arrestin-bound complexes can desensitize certain signalling cascades, such as those mediated through Gα, and also propagate distinct signalling processes through scaffolding various precursors to pathways resulting in MAPK, c-Src and Akt activation (DeWire *et al*., 2007). Association with β-arrestin also influences compartmentalization of complexes in the cellular milieu including internalization, recycling and degradation through their ability to bind other structural and regulatory proteins (Moore *et al*., 2006). Within these complexes, ubiquitination of both receptors and β-arrestins introduces another level of regulation that can influence compartmentalization, trafficking and signalling properties of the complex (Becuwe *et al*., 2012).

Recruitment and stability of GPCR-β-arrestin interactions is dependent on the affinity of β-arrestin for the GPCR. This typically occurs through phosphorylation of serine and threonine residue clusters in the C-terminal tail of GPCRs by GPCR kinases (GRKs). Phosphorylation of these residues provides the necessary chemical energy to promote high affinity interaction between receptors and β-arrestins (Gurevich and Gurevich, 2006). The presence or absence of clusters of these residues can influence the temporal stability of GPCR-arrestin interactions. This has been characterized for a number of GPCRs including the β_2_-adrenoceptors, vasopressin V_1A_ and V_2_ receptors, μ- and δ-opioid receptors, thyrotropin-releasing hormone TRH_1_ receptors, angiotensin II AT_1A_ receptors and dopamine D_2_ receptors and may broadly promote separation of GPCRs towards different downstream trafficking and signalling outcomes based upon their relative degree of arrestin association (Oakley *et al*., 1999; 2000; 2001[Bibr b17],[Bibr b18],[Bibr b19]; Kafi *et al*., 2011).

Using BRET techniques, we have shown that both OX_1_ and OX_2_ receptors display relatively high stability in forming complexes with both β-arrestins (Dalrymple *et al*., 2011). However, in contrast to other receptors that display stable arrestin association, tangible differences between the BRET kinetics of the OX_1_ and OX_2_ receptor-β-arrestin complexes were only observed upon prolonged measurement. Extended BRET (eBRET) assays displayed kinetic profiles between β-arrestin, ubiquitin and OX_1_ receptors that were more transient over a period of 4 h of orexin A stimulation, compared with profiles between β-arrestin, OX_2_ receptors and ubiquitin which exhibited a more robust and stable kinetic profile (Dalrymple *et al*., 2011). In addition, temporal ERK1/2 phosphorylation could be similarly subtype specifically distinguished between the orexin receptors over extended periods of agonist stimulation. These long-term departures of orexin receptor-β-arrestin BRET kinetics suggest a mechanism for differential orexin receptor subtype function.

To gain an insight into such possible mechanisms involved in functional orexin receptor subtype distinctions, the contribution of the C-terminal tail of OX_2_ receptors was investigated through bulk substitution and site-specific mutagenesis. Previous studies that investigated molecular determinants involved in GPCR-β-arrestin interactions revealed the nature of serine/threonine cluster sites primarily phosphorylated by GRKs (Oakley *et al*., 1999; 2000; 2001[Bibr b17],[Bibr b18],[Bibr b19]) and specifically for OX_1_ (Milasta *et al*., 2005). Using these principles, the contributions of both the entire C-terminal tail as well as three putative GRK phosphorylation sites within the C-terminal tail of OX_2_ receptors were assessed to investigate the formation and stability of OX_2_ receptor-arrestin-ubiquitin complexes and to demonstrate key structural features that defined subtype-specific functions of orexin receptors.

## Methods

### cDNA constructs and mutagenesis

Haemagglutinin (HA)-tagged human OX_1_ receptor cDNA was from Missouri S&T Resource Center (Rolla, MO, USA; Cat. No. HCR010TN00). Wild-type human OX_2_ receptor cDNA was kindly provided by M. Yanagisawa (Howard Hughes Medical Institute, Dallas, TX, USA; Accession No. NM_001526). β-arrestin1 and β-arrestin2 cDNAs were from RZPD GenomeCube, Berlin, Germany. OX_2_ multiple point mutants were generated in the C-terminus using site-directed mutagenesis to replace serine and threonine residues with alanine. cDNA mutations in OX_2_ receptors are as follows: ‘Δ399’ (a1195g, a1198g, g1199c, a1201g, a1207g, g1208c) resulting in amino acid mutations T399A, S400A, T401A and S403A; ‘Δ406’ (t1216g, a1222g, a1225g) resulting in amino acid mutations S406A, T408A and T409A; ‘Δ427’ (a1279g, a1282g, g1283c, a1288g, g1289c, a1291g) resulting in amino acid mutations T427A, S428A, S430A and T431A, and a single point mutation (g1204c) ‘E402Q’. The chimeric receptor, OX_1_ receptor with the C-terminal tail of OX_2_ receptor (OX_1_ctOX_2_), was generated by cleaving OX_1_ receptors with the PflM1 restriction enzyme, and introducing a PflM1 restriction site in OX_2_ receptors at the equivalent site by PCR mutagenesis. The C-terminal fragment of OX_2_ receptor (bases 1054–1335) was ligated with the N-terminal fragment of OX_1_ receptor (bases 1–1035). To generate cDNA encoding for BRET fusion proteins, sequences were PCR-amplified and subcloned into pcDNA3.1(+) backbone vectors containing Venus yellow fluorescent protein kindly provided by Atsushi Miyawaki (RIKEN Brain Science Institute, Wako City, Japan) or *Renilla* luciferase 8 (Rluc8) cDNA kindly provided by Andreas Loening and Sanjiv Gambhir (Stanford University, Stanford, CA, USA) as described previously for other GPCR constructs (Kocan *et al*., 2008). The stop codon between the sequences was removed to generate constructs capable of being translated into fusion proteins upon transfection, as described previously (Pfleger and Eidne, 2003; Jaeger *et al*., 2010), and all receptors were HA-tagged. BRET-tagged Kras constructs were generously provided by Nevin Lambert, Georgia Regents University, Augusta, GA, USA, and their use has been described previously (Lan *et al*., 2011; 2012[Bibr b12],[Bibr b13]; Jensen *et al*., 2013). cDNA encoding Venus-ubiquitin fusion proteins has been described previously (Dalrymple *et al*., 2011). Fusion cDNA constructs were verified by BDT labelling and capillary separation on an AB3730xl sequencer (Australian Genome Research Facility, Brisbane, Australia) and compared with published sequence data.

### Test systems

#### Cell culture and transfection

HEK293FT cells (Life Technologies, Mulgrave, Vic., Australia) were maintained at 37°C in 5% CO_2_ and complete media (DMEM) containing 0.3 mg mL^−1^ glutamine, 100 IU mL^−1^ penicillin, and 100 μg mL^−1^ streptomycin (Life Technologies) supplemented with 10% fetal calf serum (FCS, Life Technologies). HEK293FT media also contained geneticin (G418; 400 μg mL^−1^; Life Technologies). Transfections were carried out 24 h after cell seeding using GeneJuice (Novagen, Merck KGaA, Darmstadt, Germany) according to manufacturer's instructions.

### Measurements

#### Inositol phosphate assays

Inositol phosphate was measured through determination of inositol-1-phosphate accumulation and performed in 96-well microplates using the IP-One HTRF® assay (CisBio Bioassays, Bagnol sur Ceze, France) according to manufacturer's instructions, as described previously (Mustafa *et al*., 2012). Cells were stimulated with orexin A ligand for 30 min at 37°C before addition of measurement reagents. The assay was incubated for 2 h at room temperature and terbium cryptate fluorescence and time-resolved fluorescence resonance energy transfer signals were measured at 620 and 665 nm, respectively, 60 μs after excitation at 340 nm using the EnVision 2102 multilabel plate reader (PerkinElmer Life Sciences, Melbourne, Vic., Australia).

#### BRET assays

HEK293FT cells transfected 48 h earlier were harvested and prepared as described previously in 96-well plates (Nunc, Thermo Scientific, Waltham, MA, USA) (Dalrymple *et al*., 2011). Cells for eBRET assays were resuspended in HEPES-buffered (25 mM) phenol-red free DMEM with 5% FCS to maintain viability. EnduRen™ substrate (Promega, Madison, WI, USA) was added to each well at a final concentration of 60 μM. Cells were maintained for 2 h at 37°C, 5% CO_2_ for the cell permeable substrate to reach equilibrium. Samples were read sequentially using a VICTOR Light™ 1420 luminescence counter (PerkinElmer Life Sciences) with 400–475 nm (‘donor emission’) and 520–540 nm (‘acceptor emission’) filters, except for Figures 2B, 6A and 6B. For these figures, data were generated using a POLARstar Omega (BMG Labtech, Mornington, Vic., Australia) with 460–490 nm (‘donor emission’) and 520–550 nm (‘acceptor emission’) filters. eBRET kinetics were measured for approximately 30 min to obtain a basal signal. Cells were then treated with vehicle or ligand and read continuously for several hours. Ligand-induced BRET signals were calculated by subtracting the ratio of ‘acceptor emission’ over the ‘donor emission’ for a vehicle-treated cell sample containing both the Rluc8 and Venus fusion proteins from the same ratio for a second aliquot of the same cells that was treated with ligand as described previously (Kocan *et al*., 2008). The final pretreatment measurement is presented at the zero time point (time of ligand or vehicle addition). BRET signals for assays in the presence of BRET-tagged Kras were calculated by subtracting the ratio of ‘acceptor emission’ over the ‘donor emission’ for a cell sample containing only the Rluc8 fusion protein from the same ratio of a second aliquot of cells containing both the Rluc8 and Venus fusion proteins. For these assays, 15 measurements were taken over the course of 25 min and averaged for each construct.

#### Data analysis

Data were presented and analysed using Prism 6 graphing software (GraphPad, San Diego, CA, USA). Sigmoidal dose-response curves were fitted using non-linear regression. Statistical significance for dose-response and eBRET kinetic data was determined using one-way anova and Tukey's multiple comparison *post hoc* tests.

### Materials

Orexin A was sourced from the American Peptide Company (Sunnyvale, CA, USA).

## Results

### Investigation of an OX_1_ receptor chimera with C-terminal tail of OX_2_ receptor

**Figure 1:**
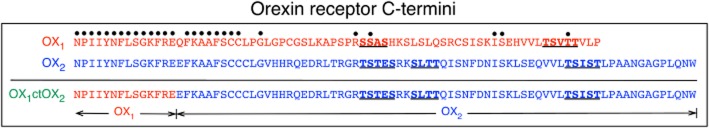
Diagrammatic representation of the primary amino acid structures of the C-termini of OX_1_, OX_2_ and the OX_1_ctOX_2_ mutant receptors. Dots above residues indicate identical amino acids in OX_1_ and OX_2_ receptors when aligned from the *NPIIY* motif at the end of transmembrane domain 7. The OX_1_ctOX_2_ mutant contains amino acids 1–367 of the OX_1_ receptor and amino acids 374–444 of the OX_2_ receptor, as indicated. Underlined, bold residues are putative GRK phosphorylation cluster sites in OX_1_ and OX_2_ receptors.

**Figure 2:**
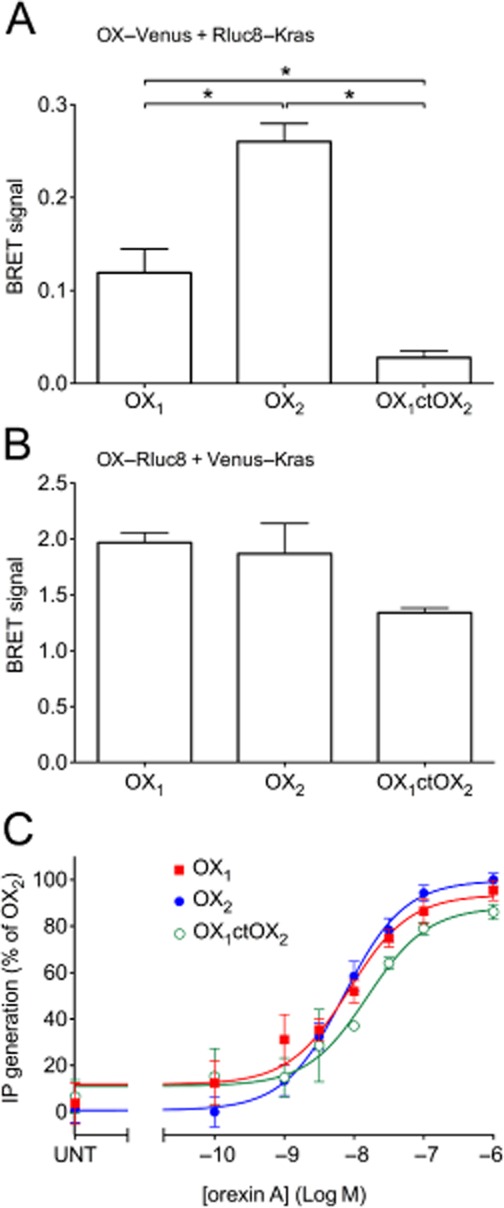
BRET proximity data between Rluc8-Kras and Venus-tagged OX_1_, OX_2_, OX_1_ctOX_2_ receptors (A), or Venus-Kras and Rluc8-tagged OX_1_, OX_2_ and OX_1_ctOX_2_ receptors (B). Concentration-response data of inositol phosphate production for OX_1_, OX_2_ and OX_1_ctOX_2_ receptors. HEK293FT cells were transiently transfected with C-terminally Venus-tagged OX_1_, OX_2_ or OX_1_ctOX_2_ receptors and treated with orexin A at concentrations shown (C). pEC_50_ values were as follows: 8.07 ± 0.15 (OX_1_), 8.15 ± 0.09 (OX_2_) and 7.81 ± 0.17 (OX_1_ctOX_2_). These values were not significantly different from each other (anova; *P* = 0.28). Significant differences in maximal efficacy were also not observed (anova; *P* = 0.052). Values for maximal efficacy of OX_1_ and OX_1_ctOX_2_ receptors were 95.4 ± 4.4% and 86.1 ± 3.3% of OX_2_ receptor respectively. ‘UNT’ refers to untreated cells transfected with each OX receptor construct (C). Data are expressed as mean ± SEM of at least three independent experiments. * *P* < 0.05, significantly different, as indicated.

**Figure 3:**
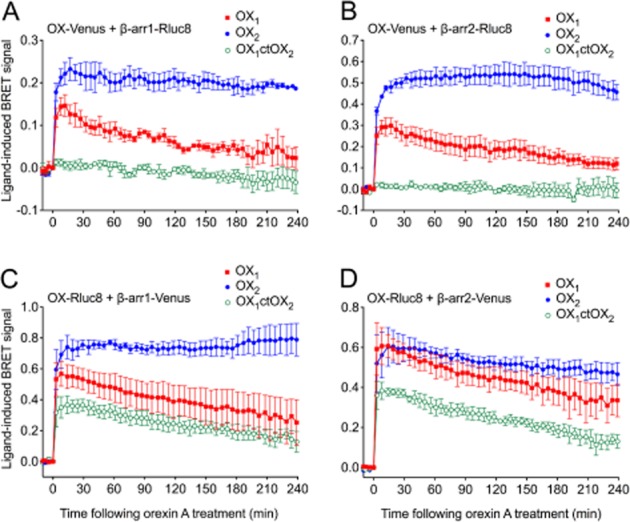
eBRET kinetic data for OX_1_, OX_2_ and OX_1_ctOX_2_ receptors. HEK293FT cells transiently transfected with C-terminally Venus-tagged receptors and Rluc8-tagged β-arrestin1 (A) or β-arrestin2 (B), or C-terminally Rluc8-tagged receptors and Venus-tagged β-arrestin1 (C) or β-arrestin2 (D) were treated with 0.6 μM orexin A. Data are presented as mean ± SEM of three independent experiments.

Based on the findings of our previous study (Dalrymple *et al*., 2011), we hypothesized that a chimeric receptor involving the replacement of the C-terminal region of OX_1_ receptor with that of OX_2_ receptor (OX_1_ctOX_2_; Figure 1) would result in a gain-of-function with respect to arrestin binding stability. BRET data indicating proximity between the cell surface marker, Kras, and OX_1_, OX_2_ receptors and the OX_1_ctOX_2_ mutant (Figure 2A, B) revealed that a decreased level of the mutant OX_1_ctOX_2_ appears to be present at the cell surface, despite maintaining potency and maximal efficacy for orexin A-stimulated inositol phosphate production that was not different from that of either OX_1_ or OX_2_ receptors (Figure 2C). These data indicate that although G protein-mediated functions with regard to Gα_q_-coupling were not sensitive to this bulk alteration, this mutant was impaired in being suitably targeted to the cell surface compared to OX_1_ and OX_2_ receptors (although presumably not enough to deplete the receptor reserve given the lack of effect on inositol phosphate signalling). Ligand-induced BRET assays for β-arrestin proximity were carried out in both BRET-tag orientations for comparison (Figure 3). Importantly, for both BRET-tag orientations, OX_2_ receptors provided a more stable BRET signal due to β-arrestin proximity, than observed with OX_1_ receptors (Figure 3), consistent with our previous findings using EGFP-tagged OX receptors and Rluc-tagged β-arrestins (Dalrymple *et al*., 2011). The Venus-tagged receptor orientation resulted in a very low ligand-induced BRET signal for the mutant compared to either wild-type OX receptor subtype (Figure 3A, B). In contrast, the Rluc8-tagged receptor orientation resulted in substantially greater BRET signals between the OX_1_ctOX_2_ mutant and both β-arrestin1 and 2 (Figure 3C, D). Interestingly, this OX_1_ctOX_2_ mutant displays similar kinetics to OX_1_ receptor, its profile being less stable over the 4 h measurement period compared to that of OX_2_ receptor (Figure 3C, D).

### Effect of serine/threonine clusters on OX_2_ receptor-arrestin proximity

**Figure 4:**
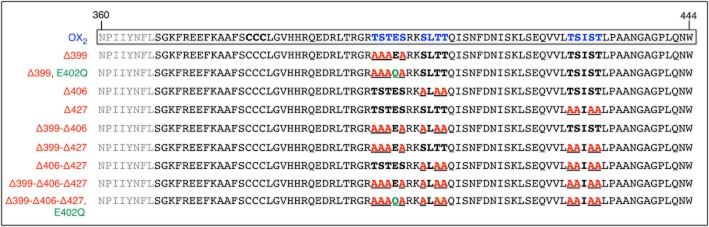
Diagrammatic representation of OX_2_ and each of the OX_2_ C-terminal mutant receptors used in this study in terms of primary amino acid structure. Amino acids 360–444 corresponding to the C-terminal tail region of the OX_2_ receptor are shown. Residues indicated in bold are in the serine/threonine (S/T) clusters that were assessed as putative GRK phosphorylation sites (Oakley *et al*., 2001). Underlined bold residues (in red) indicate amino acids within each of the clusters that were mutated to alanine. Additionally, glutamate 402 was mutated to glutamine as indicated (in green).

**Figure 5:**
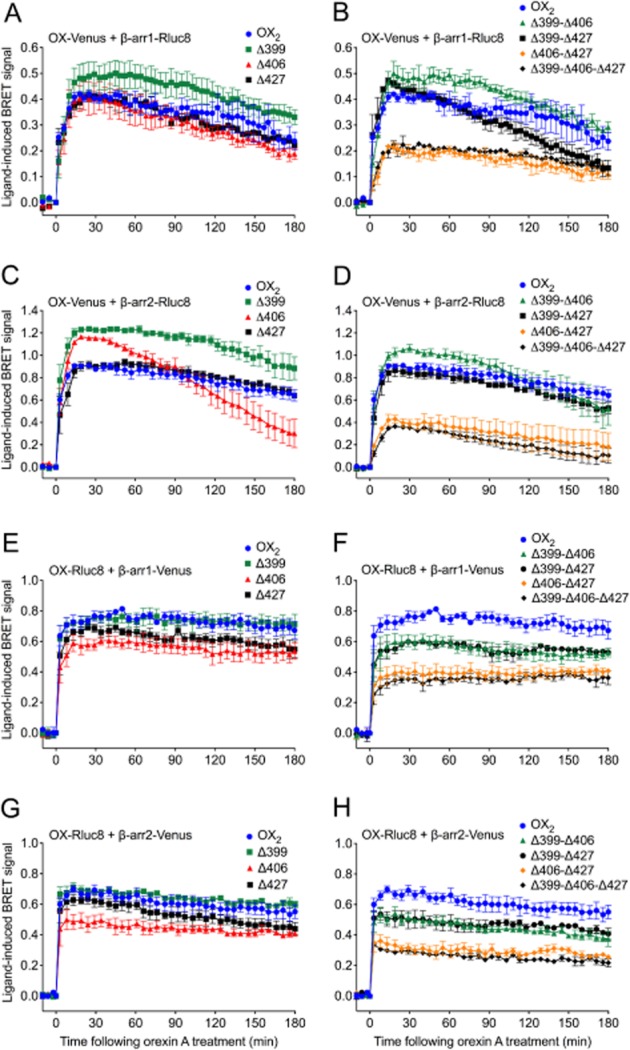
eBRET data indicating proximity between OX_2_ or OX_2_ C-terminal tail mutant receptors with β-arrestin1 or 2. HEK293FT cells were transiently transfected with either C-terminally Venus-tagged (A-D), or Rluc8-tagged (E-H) OX_2_ or each of the single (A, C, E, G) or double/triple (B, D, F, H) C-terminal OX_2_ mutant receptors in the presence of either Rluc8-tagged β-arrestin1 (A, B) or β-arrestin2 (C, D), or Venus-tagged β-arrestin1 (E, F) or β-arrestin2 (G, H). The zero time point indicates the point at which 0.6 μM orexin A was added. Data are presented as mean ± SEM of three independent experiments.

To gain more specific insights into the mechanism of orexin receptor-arrestin interaction, serine and threonine residues in defined clusters in the C-terminal tail were mutated to alanine, generating a series of OX_2_ receptor mutants (Figure 4). BRET proximity time course assays were subsequently carried out between these mutants and β-arrestin1 or 2 in both BRET-tag orientations (Figure 5). Mutation of a single cluster in isolation, except for the Δ406, did not notably reduce the strength of the ligand-induced BRET signal compared to wild-type OX_2_ receptors (Figure 5A, C, E, G). Interestingly with β-arrestin2, the BRET signal for the Δ406 mutant displays a dramatic change in BRET kinetics when Venus-tagged (Figure 5C). Rluc8-tagged OX_2_ receptors and each of the single cluster mutants displayed greater BRET signal stability. Nevertheless, the Δ406 mutant appears to display a marginally suppressed BRET signal compared to the other single mutants (Figure 5E, G). The data from both the Venus-tagged and Rluc8-tagged double and triple mutants indicate that the Δ406-Δ427 and Δ399-Δ406-Δ427 mutants display a substantially lowered BRET signal compared to wild-type OX_2_ receptors (Figure 5B, D, F, H). Interestingly, the Rluc8-tagged receptors display a hierarchy in BRET signal magnitude. A ‘step-wise’ reduction in BRET signal was observed with both β-arrestin subtypes, dependent upon the presence of either the Δ406 or Δ427 cluster, and appears to be additive independently of the Δ399 mutation (Figure 5F, H). These results should also be considered in the context of relative receptor expression levels at the plasma membrane (see below).

### Effect of serine/threonine cluster mutations on cell surface expression and inositol phosphate production

**Figure 6:**
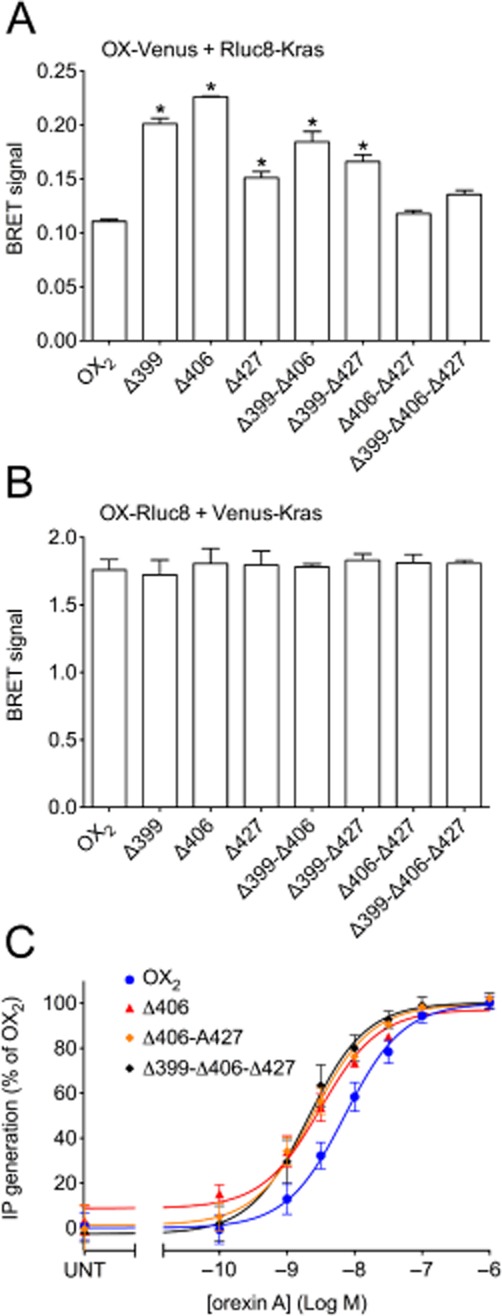
eBRET data indicating proximity between Rluc8-Kras and Venus-tagged OX_2_ wild-type and mutant receptors (A), or Venus-Kras and Rluc8-tagged OX_2_ wild-type and mutant receptors (B). Inositol phosphate concentration-response data for OX_2_ wild-type and mutant receptors. Transiently transfected HEK293FT cells with C-terminally Venus-tagged wild-type OX_2_ or OX_2_ mutant receptors (Δ406, Δ406-Δ427 or Δ399-Δ406-Δ427) were treated with doses of orexin A as shown (C). pEC_50_ values were: 8.15 ± 0.09 (OX_2_); 8.51 ± 0.08 (Δ406); 8.62 ± 0.09 (Δ406-Δ427) and 8.69 ± 0.10 (Δ399-Δ406-Δ427). Values for maximal efficacy as a percentage of OX_2_ receptors are as follows: 101.2 ± 0.2 (Δ406), 102.1 ± 1.7 (Δ406-Δ427), 101.1 ± 3.3 (Δ399-Δ406-Δ427). ‘UNT’ refers to untreated cells transfected with each OX receptor construct (C). Data are presented as mean ± SEM of at least three independent experiments. * *P* < 0.05, significantly different from wild-type OX_2_ receptor.

BRET proximity of OX_2_ receptors and each of the mutants with the cell surface marker Kras provides insights into their relative plasma membrane expression levels. Notably, there were no significant reductions in receptor-Kras BRET signal with either BRET-tag orientation (Figure 6A, B), indicating that reductions in receptor-arrestin BRET signals relative to wild-type were not as a consequence of reduced receptor plasma membrane expression. Interestingly, with the Venus-tagged receptors, some mutants appear to be expressed at higher levels. More specifically, it is notable that the receptor-arrestin BRET signals for Δ399 are higher than wild-type in Figure 5A and C, and the signal for Δ406 is initially higher in Figure 5C, which correlates with these mutants appearing to be expressed at substantially higher levels on the plasma membrane from the receptor-Kras BRET data. Furthermore, the Δ399-Δ406 double mutant gave the highest receptor-Kras BRET signal of the Venus-tagged double mutants, which may account for the receptor-arrestin BRET signals for this mutant being higher than observed for wild type OX_2_ receptor (Figure 5B, D). In contrast, all of the Rluc8-tagged mutants displayed similar BRET signals to wild-type OX_2_ receptor with Kras (Figure 6B). The Venus-tagged Δ406, Δ406-Δ427 and Δ399-Δ406-Δ427 mutants were also analysed and compared to wild-type Venus-tagged OX_2_ receptor to assess their ability to stimulate the turnover of inositol phosphates (Figure 6C). The potency of all of these mutants was significantly increased compared to wild-type OX_2_ receptor (*P* < 0.05). These data indicate that no loss of G protein coupling results from these mutations. Indeed, it is hypothesized that decreased desensitization of G protein-mediated signalling as a consequence of the reduced recruitment of β-arrestins contributes to this increase in G protein coupling potency.

### Comparison of receptor-arrestin interaction potency

**Figure 7:**
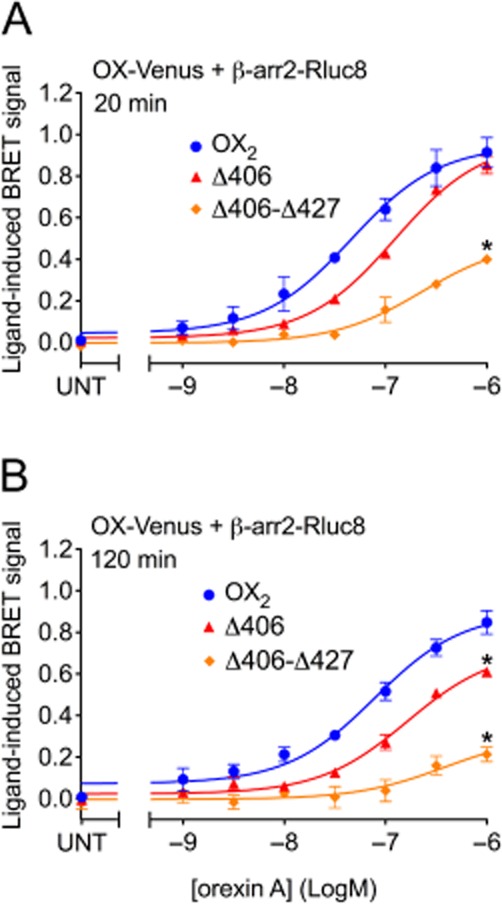
eBRET dose-response data indicating proximity between β-arrestin2 and OX_2_, or OX_2_ mutants Δ406 or Δ406-Δ427, at 20 and 120 min post-agonist stimulation. pEC_50_ values were as follows: 7.34 ± 0.11 (OX_2_), 6.91 ± 0.05 (Δ406), 6.64 ± 0.14 (Δ406-Δ427) at 20 min; 7.11 ± 0.10 (OX_2_), 6.79 ± 0.08 (Δ406), 6.42 ± 0.43 (Δ406-Δ427) at 120 min. Maximal BRET efficacy values are as follows: 0.91 ± 0.07 (OX_2_), 0.85 ± 0.04 (Δ406), 0.40 ± 0.02 (Δ406-Δ427) at 20 min; 0.85 ± 0.06 (OX_2_), 0.61 ± 0.01 (Δ406), 0.21 ± 0.03 (Δ406-Δ427) at 120 min. Data are presented as mean ± SEM of three independent experiments. * *P* < 0.05, significantly different from OX_2_ receptors.

Dose-response data of orexin A-stimulated BRET between Venus-tagged OX_2_, OX_2_ Δ406 or OX_2_ Δ406-Δ427 receptors and β-arrestin2 are shown for an early time point (20 min; Figure 7A) and a later time point (120 min; Figure 7B). The potency of β-arrestin2 recruitment to the Δ406 mutant was not reduced sufficiently to reach statistical significance compared to wild-type OX_2_ receptor (Figure 7). In contrast, the potency observed with the Δ406-Δ427 mutant was fivefold lower compared to OX_2_ receptor, and this difference was statistically significant at the earlier time point (Figure 7A; *P* < 0.05). Maximal efficacy of the Δ406-Δ427 mutant was also substantially reduced compared to both OX_2_ and the Δ406 mutant receptors (Figure 7). These findings are consistent with the eBRET kinetic data suggesting that the Δ406-Δ427 mutant is substantially impaired in its ability to recruit β-arrestins (Figure 5B, D, F, H). Interestingly, maximal efficacy of the Δ406 mutant was significantly reduced relative to the wild-type OX_2_ receptor after 120 min of orexin A stimulation (Figure 7B), but not after 20 min of stimulation (Figure 7A), consistent with the kinetic profile shown in Figure 5C.

### Investigation of glutamate as a potential phosphate mimic in the proximal serine/threonine cluster

**Figure 8:**
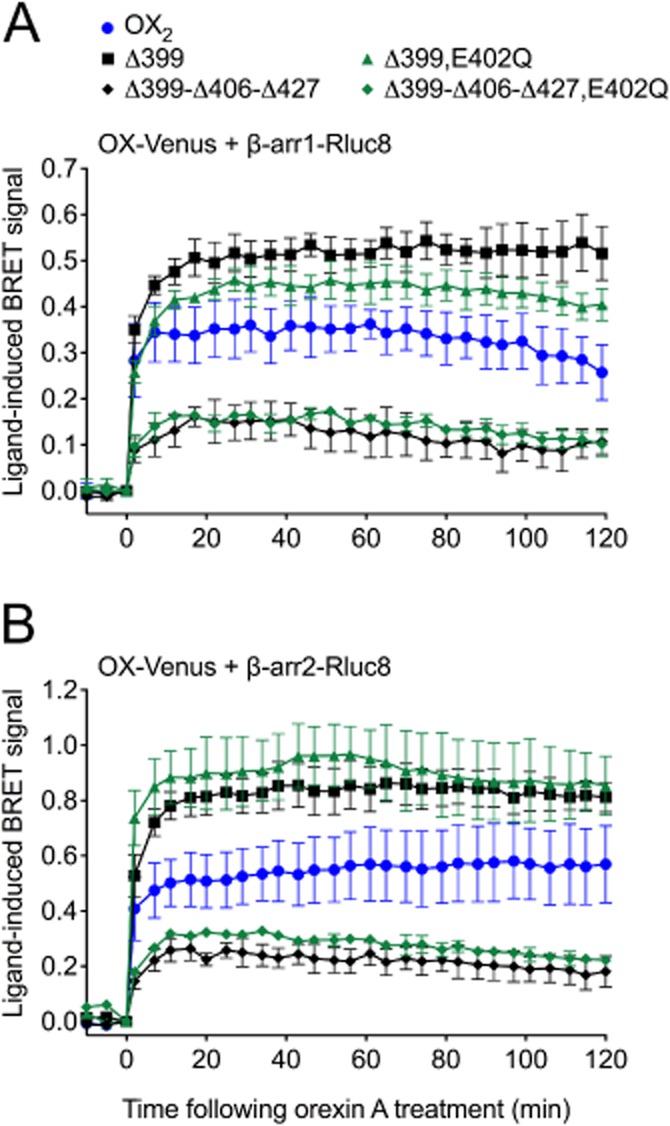
eBRET data comparing proximity between β-arrestin1 or 2 and OX_2_, OX_2_ Δ399 or OX_2_ Δ399-Δ406-Δ427 receptors, with or without the E402Q mutation. HEK293FT cells were transiently transfected with Venus-tagged OX_2_ or mutant receptors and either Rluc8-tagged β-arrestin1 (A) or β-arrestin2 (B). The zero time point indicates when 0.6 μM orexin A was added. Data are presented as mean ± SEM of three independent experiments.

To investigate the possibility that the negatively charged glutamate residue in the Δ399 cluster (E402) may have a contributing effect on the stability of the interaction with β-arrestin, a comparison was made between the mutants Δ399 and Δ399-E402Q, and between Δ399-Δ406-Δ427 and Δ399-Δ406-Δ427-E402Q (Figure 4). However, no notable deviations in BRET signal kinetics or magnitude were observed as a consequence of this additional mutation (Figure 8).

### Ubiquitin-arrestin proximity in the presence of OX_2_ receptor and serine/threonine cluster mutants

**Figure 9:**
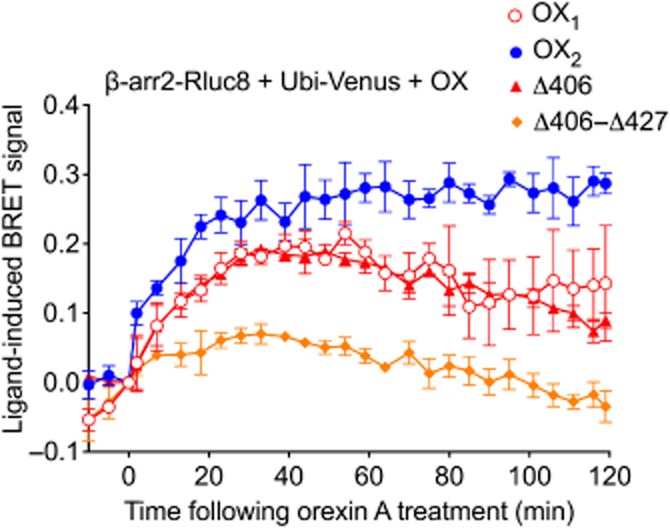
eBRET data indicating proximity between ubiquitin and β-arrestin2 in the presence of wild-type OX_1_, OX_2_ or OX_2_ mutant receptors. HEK293FT cells were transiently transfected with N-terminally Venus-tagged ubiquitin, C-terminally Rluc8-tagged β-arrestin2 and non-BRET-tagged OX_1_, OX_2_, OX_2_ Δ406 or OX_2_ Δ406-Δ427 receptors. The zero time point indicates when 0.6 μM orexin A was added. Data are presented as mean ± SEM of three independent experiments.

In an alternate BRET configuration, the orexin receptor complex was observed through the measurement of proximity between β-arrestin2 and ubiquitin in the presence of non-BRET-tagged receptors to reveal further properties of these mutant OX_2_ receptor complexes. BRET proximity assays revealed a robust and stable signal for OX_2_ and a less sustained kinetic signal for OX_1_ receptors (Figure 9), as observed previously (Dalrymple *et al*., 2011). In contrast, and consistent with the receptor-arrestin proximity (Figure 5) and dose-response data (Figure 7), diminished arrestin-ubiquitin proximity was observed in the presence of the stimulated OX_2_ Δ406-Δ427 mutant, with the BRET signal returning to baseline levels sooner than with OX_1_ or OX_2_ receptors. Interestingly, the kinetic BRET profile observed in the presence of OX_2_ Δ406 mutant receptors was essentially identical to that observed with OX_1_ receptors (Figure 9).

## Discussion and conclusions

We have previously shown that the two orexin receptor subtypes display diverging BRET kinetic profiles when forming β-arrestin and ubiquitin complexes, with differences in cellular localization and signalling also being observed (Dalrymple *et al*., 2011). OX_2_ receptors displayed greater stability over time when forming β-arrestin complexes compared with OX_1_ receptors, along with a sustained ability to maintain phosphorylated ERK1/2 while being unable to rapidly recycle upon internalization. Prior to that study, OX_1_ receptors had been found to colocalize with β-arrestin1 upon orexin A stimulation using confocal microscopy (Evans *et al*., 2001) and specific sites in these receptors were shown to be involved in β-arrestin interaction (Milasta *et al*., 2005). Our aim was therefore to establish the molecular determinants responsible for OX_2_ receptor interactions with β-arrestin and to understand the molecular basis for the differences in OX_1_ and OX_2_ receptor function that we had observed, in terms of receptor-arrestin-ubiquitin complex stability.

The OX_1_ctOX_2_ chimera (Figure 1) was generated to investigate whether the C-terminal tail of OX_2_ receptors could bestow greater stability to the OX_1_ receptor-β-arrestin complex. However, ligand-induced BRET assays indicated that BRET-tag orientation made a substantial difference to our ability to detect a β-arrestin proximity BRET signal specifically for this chimera, in contrast to the wild-type receptors (Figure 3). The cell surface expression of the Venus-tagged OX_1_ctOX_2_ chimera was significantly reduced, compared with that of OX_1_ and OX_2_ receptors (Figure 2A), which may contribute to the reduction in β-arrestin proximity BRET signal observed. However, as the inositol phosphate generation was not affected (Figure 2C), it is unlikely that cell surface expression alone was responsible for the almost complete abolition of this BRET signal. Indeed, this finding implies that the conformation of the OX_2_ C-terminal tail is different (and therefore orients the BRET-tag differently) when attached to the rest of the OX_1_ receptor, compared with the conformation when attached to the rest of the OX_2_ receptor.

In contrast, although the cell surface expression may have been slightly reduced with the Rluc8-tagged OX_1_ctOX_2_ chimera (Figure 2B), a BRET signal was observed for proximity to the Venus-tagged β-arrestins (Figure 3C, D). Notably, the resultant kinetic profile of Rluc8-tagged OX_1_ctOX_2_ was similar to that of OX_1_ and not OX_2_ receptors. These findings indicate that the primary structure of the OX C-terminal tail is not wholly responsible for determining the stability of the interaction with β-arrestins.

OX_1_ and OX_2_ receptors share significant homology in primary structure except for the N-terminus, distal region of the C-terminal tail and the third intracellular loop (ICL3) (Sakurai *et al*., 1998; Voisin *et al*., 2003). There are very few differences in ICL1 and 2, with those present not likely to have a major effect on β-arrestin recruitment. Regarding ICL3, the only putative high affinity GRK phosphorylation site is conserved (T250, T251, S252 in OX_1_ and T258, S259, S260 in OX_2_) and is in the proximal region of the loop that is itself highly conserved. Beyond this, OX_1_ ICL3 has two serines and a threonine spread through the loop, whereas OX_2_ ICL3 has three serines and two threonines, again not clustered. Therefore, from the perspective of primary structure, there are no obvious differences between the ICL3 of OX_1_ and OX_2_ receptors in terms of high affinity GRK phosphorylation sites. However, it is likely that the conformation of ICL3 differs between the receptor subtypes and how configuration of this with the C-terminus influences high affinity β-arrestin interaction is certainly worthy of future investigation.

It is our hypothesis that the lack of gain of function observed with the OX_1_ctOX_2_ mutant with respect to β-arrestin binding is due to disruption of secondary or tertiary structure, within or between the intracellular domains. This indicates that appropriate GRK phosphorylation sites need to be not only present, but correctly positioned and orientated for both phosphorylation and β-arrestin interaction. Therefore, although there are notable examples of C-terminal tail chimeras adopting the characteristics of the substituted C-terminal tail, such as β_2_-adrenoceptor-V_2_-tail or V_2_-β_2_-adrenoceptor tail chimeras (Oakley *et al*., 1999; Shenoy and Lefkowitz, 2003; Tohgo *et al*., 2003) as well as chimeras of β_2_-adrenoceptor-AT_1A_ (Anborgh *et al*., 2000) and NK_1_-PAR2 receptors (Pal *et al*., 2012), our data show that this is not always the case. Indeed, we have previously published work investigating the effect of extracellular loop substitution on ligand binding and signalling properties of the gonadotrophin-releasing hormone receptor (Pfleger *et al*., 2008), where interactions between loops appeared to play a role. It is likely that similar interactions between the intracellular loops and C-terminal tail are involved in configuring intracellular binding sites for β-arrestin. Therefore, although the C-terminal tail of OX_2_ receptors may function well in the spatial context of the rest of the OX_2_ receptor, it may not when set amongst the intracellular loops of the OX_1_ receptor. It is also possible that homo- or heteromerization could play a role.

To further elucidate the molecular determinants of orexin receptor-arrestin interactions, three serine/threonine clusters in the C-terminal tail of OX_2_ receptors were analysed for their ability to affect OX_2_ receptor-β-arrestin binding strength and stability (Figure 4). The BRET-tag orientation of the OX receptor constructs appears to have some influence on their relative cell surface expression levels (Figure 6A, B) and general stability of the kinetic profiles (Figure 5), however, taking this into account, the overall effects of the mutations relative to wild-type are largely consistent, regardless of BRET-tag orientation (Figure 5). Mutation of any of the cluster sites in isolation had little detrimental impact on the initial strength of the receptor-arrestin interaction, with the possible exception of the Δ406 mutant with Rluc8-tagged receptors. This contrasts with receptors such as AT_1A_ and oxytocin receptors that have a similar complement of phosphorylation clusters, but only require mutation of a single cluster to significantly disturb arrestin translocation (Oakley *et al*., 2001).

As GRK phosphorylation increases receptor affinity for β-arrestins as a consequence of introducing negative charge, it was postulated that the negatively charged glutamate within the 399 cluster could act as a phosphate mimic, as described previously (Gurevich and Gurevich, 2006). However, our data from the Δ399-E402Q mutant compared to Δ399 (and indeed the triple cluster mutant with and without E402Q) provide evidence against any such role for this residue (Figure 8).

**Figure 10:**
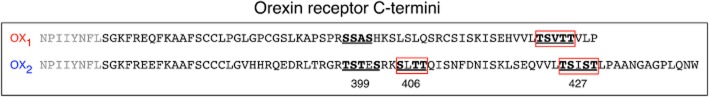
Diagram summarizing the apparently critical putative GRK phosphorylation sites in the C-terminal tail of OX_1_ and OX_2_ receptors. Underlined residues indicate the clusters that were examined for OX_1_ receptors previously (Milasta *et al*., 2005) and for OX_2_ receptors in this study. The boxed residues indicate clusters that had a notable effect on β-arrestin-mediated recruitment/colocalization with OX_1_ in the work by Milasta *et al*. (2005), and with OX_2_ receptors in the current study. Note that from our data, mutation of the 406 cluster in OX_2_ receptors had the most influence on the receptor-arrestin-ubiquitin complex over time, but mutation of both 406 and 427 clusters was required to substantially reduce the initial strength of complex formation.

A previous study revealed that mutation of the single serine/threonine cluster in the distal end of the C-terminal tail of OX_1_ receptors severely impaired arrestin translocation, but surprisingly mutation of the proximal cluster had little effect (Milasta *et al*., 2005). Upon comparison of the primary structures of OX_1_ and OX_2_ receptors, the serine/threonine clusters at 399 and 427 in OX_2_ receptor are similarly present at corresponding sites in OX_1_ receptor (Figure 10). However, an additional cluster present in OX_2_ receptors at position 406 is absent in the same corresponding region of OX_1_ receptors (Figure 10). Our findings indicate that mutation of the 406 serine/threonine cluster has the greatest effect on destabilizing the OX_2_ receptor-β-arrestin interaction, this being particularly clear with Venus-tagged receptor and Rluc8-tagged β-arrestin 2 (Figures 5C and 7). Indeed, this profile exhibits similarities to that observed in our previous study with OX_1_ receptors (Dalrymple *et al*., 2011). This alteration in kinetics is also demonstrated through arrestin-ubiquitin kinetics where the profiles of OX_2_ Δ406 and OX_1_ receptors overlap (Figure 9), in contrast to the separation in β-arrestin-ubiquitin kinetics observed for the wild-type receptors (Dalrymple *et al*., 2011). These data indicate that, in addition to the potential role of C-terminus/ICL3 configuration discussed above, the 406 cluster may contribute to the differentiation of the orexin receptor subtypes, by conferring upon OX_2_ receptors the ability to form more stable complexes with β-arrestin and ubiquitin.

Two of the three hypothesized GRK phosphorylation cluster sites, 406 and 427, appear to be involved in achieving stable OX_2_ receptor-β-arrestin complexes. Mutation of these clusters in combination appears to render substantial loss-of-function with regard to β-arrestin interaction strength, despite no apparent reduction in cell surface expression as determined by assessment of proximity to the Kras cell surface marker. These findings imply a degree of redundancy, and hint at another potential molecular mechanism to explain the previously observed difference in receptor-arrestin-ubiquitin complex stability with OX_1_ compared to OX_2_ receptors (Dalrymple *et al*., 2011). As a result of our findings, we propose a simplistic model that may help to explain some of the complex stability differences, particularly as observed in Figures 5C, 7 and 9. This model would suggest that OX_1_ receptors are phosphorylated on the distal serine/threonine cluster, as reported by Milasta *et al*. (2005), but OX_2_ receptors are phosphorylated on the two clusters that we have designated 406 and 427 (Figure 10). Perhaps only one of these sites in the OX_2_ receptor requires phosphorylation in order for the receptor to adopt a high-affinity state for arrestin binding. As the receptors are internalized, it is hypothesized that dephosphorylation of the distal serine/threonine cluster of OX_1_ receptor switches the receptor to a lower-affinity state for arrestin binding, whereas OX_2_ receptor requires both sites to be dephosphorylated for this to occur. This may then extend the time during which individual receptors remain in the high-affinity state for arrestin binding, which is consistent with the OX_2_ receptor-arrestin-ubiquitin complex being more stable over time and OX_2_ receptors recycling more slowly to the plasma membrane as a consequence (Dalrymple *et al*., 2011). This is of course not the only possible explanation, and our findings with the C-terminal chimera indicate a role for other parts of the intracellular domain as well, as discussed above.

This study therefore provides fundamental insights into the molecular determinants that govern orexin receptor subtype-specific arrestin-ubiquitin complex formation and stability. Our findings, and the conceptual models they have elicited, provide further potential molecular explanations for our previous observations (Dalrymple *et al*., 2011) and enable us to begin making key correlations between structure and function.

## Abbreviations

eBRET: extended BRET
FCS: fetal calf serum
GRK: GPCR kinase
HA: hemagglutinin
ICL: intracellular loop
OX_1_ctOX_2_: chimera of OX_1_ receptor with OX_2_ receptor C-terminal tail
Rluc8: *Renilla luciferase 8*

## References

[1] Anborgh PH, Seachrist JL, Dale LB, Ferguson SS (2000). Receptor/beta-arrestin complex formation and the differential trafficking and resensitization of beta2-adrenergic and angiotensin II type 1A receptors. Mol Endocrinol.

[2] Becuwe M, Herrador A, Haguenauer-Tsapis R, Vincent O, Leon S (2012). Ubiquitin-mediated regulation of endocytosis by proteins of the arrestin family. Biochem Res Int.

[3] Dalrymple MB, Jaeger WC, Eidne KA, Pfleger KD (2011). Temporal profiling of orexin receptor-arrestin-ubiquitin complexes reveals differences between receptor subtypes. J Biol Chem.

[4] DeWire S, Ahn S, Lefkowitz R, Shenoy S (2007). Beta-arrestins and cell signaling. Annu Rev Physiol.

[5] Evans NA, Groarke DA, Warrack J, Greenwood CJ, Dodgson K, Milligan G (2001). Visualizing differences in ligand-induced beta-arrestin-GFP interactions and trafficking between three recently characterized G protein-coupled receptors. J Neurochem.

[6] Gurevich VV, Gurevich EV (2006). The structural basis of arrestin-mediated regulation of G-protein-coupled receptors. Pharmacol Ther.

[7] Jaeger WC, Pfleger KDG, Eidne KA, Poyner DR, Wheatley M (2010). Monitoring GPCR–protein complexes using bioluminescence resonance energy transfer. G Protein-Coupled Receptors.

[8] Jensen DD, Godfrey CB, Niklas C, Canals M, Kocan M, Poole DP (2013). The bile acid receptor TGR5 does not interact with beta-arrestins or traffic to endosomes but transmits sustained signals from plasma membrane rafts. J Biol Chem.

[9] Kafi AKM, Hattori M, Misawa N, Ozawa T (2011). Dual-color bioluminescence analysis for quantitatively monitoring G-protein-coupled receptor and β-arrestin interactions. Pharmaceuticals.

[10] Kim AK, Brown RM, Lawrence AJ (2012). The role of orexins/hypocretins in alcohol use and abuse: an appetitive-reward relationship. Front Behav Neurosci.

[11] Kocan M, See HB, Seeber RM, Eidne KA, Pfleger KD (2008). Demonstration of improvements to the bioluminescence resonance energy transfer (BRET) technology for the monitoring of G protein-coupled receptors in live cells. J Biomol Screen.

[b12] Lan TH, Kuravi S, Lambert NA (2011). Internalization dissociates beta2-adrenergic receptors. PLoS One.

[b13] Lan TH, Liu Q, Li C, Wu G, Lambert NA (2012). Sensitive and high resolution localization and tracking of membrane proteins in live cells with BRET. Traffic.

[14] Milasta S, Evans NA, Ormiston L, Wilson S, Lefkowitz RJ, Milligan G (2005). The sustainability of interactions between the orexin-1 receptor and beta-arrestin-2 is defined by a single C-terminal cluster of hydroxy amino acids and modulates the kinetics of ERK MAPK regulation. Biochem J.

[15] Moore C, Milano S, Benovic J (2006). Regulation of receptor trafficking by GRKs and arrestins. Annu Rev Physiol.

[16] Mustafa S, See HB, Seeber RM, Armstrong SP, White CW, Ventura S (2012). Identification and profiling of novel alpha1A-adrenoceptor-CXC chemokine receptor 2 heteromer. J Biol Chem.

[b17] Oakley RH, Laporte SA, Holt JA, Barak LS, Caron MG (1999). Association of beta-arrestin with G protein-coupled receptors during clathrin-mediated endocytosis dictates the profile of receptor resensitization. J Biol Chem.

[b18] Oakley RH, Laporte SA, Holt JA, Caron MG, Barak LS (2000). Differential affinities of visual arrestin, beta arrestin1, and beta arrestin2 for G protein-coupled receptors delineate two major classes of receptors. J Biol Chem.

[b19] Oakley RH, Laporte SA, Holt JA, Barak LS, Caron MG (2001). Molecular determinants underlying the formation of stable intracellular G protein-coupled receptor-beta-arrestin complexes after receptor endocytosis. J Biol Chem.

[20] Pal K, Mathur M, Kumar P, Defea K (2012). Divergent beta-arrestin-dependent signaling events are dependent upon sequences within G-protein-coupled-receptor C-termini. J Biol Chem.

[21] Pfleger KD, Eidne KA (2003). New technologies: bioluminescence resonance energy transfer (BRET) for the detection of real time interactions involving G-protein coupled receptors. Pituitary.

[22] Pfleger KD, Pawson AJ, Millar RP (2008). Changes to gonadotropin-releasing hormone (GnRH) receptor extracellular loops differentially affect GnRH analog binding and activation: evidence for distinct ligand-stabilized receptor conformations. Endocrinology.

[23] Sakurai T, Amemiya A, Ishii M, Matsuzaki I, Chemelli RM, Tanaka H (1998). Orexins and orexin receptors: a family of hypothalamic neuropeptides and G protein-coupled receptors that regulate feeding behavior. Cell.

[24] Sakurai T, Mieda M (2011). Connectomics of orexin-producing neurons: interface of systems of emotion, energy homeostasis and arousal. Trends Pharmacol Sci.

[25] Shenoy SK, Lefkowitz RJ (2003). Trafficking patterns of beta-arrestin and G protein-coupled receptors determined by the kinetics of beta-arrestin deubiquitination. J Biol Chem.

[26] Tohgo A, Choy E, Gesty-Palmer D, Pierce K, Laporte S, Oakley R (2003). The stability of the G protein-coupled receptor-beta-arrestin interaction determines the mechanism and functional consequence of ERK activation. J Biol Chem.

[27] Voisin T, Rouet-Benzineb P, Reuter N, Laburthe M (2003). Orexins and their receptors: structural aspects and role in peripheral tissues. Cell Mol Life Sci.

[28] Alexander SPH (2013). The Concise Guide to PHARMACOLOGY 2013/14: Overview. Br J Pharmacol.

